# Efficacy of treating *Helicobacter pylori* infection on seizure frequency in children with drug-resistant idiopathic generalized epilepsy: a randomized controlled trial

**DOI:** 10.1186/s13052-025-01956-2

**Published:** 2025-04-17

**Authors:** Mostafa Ashry Mohamed, Ekram A. Mahmoud, Mina S. Basily, Montaser M. Mohamed, Omar A. A. Ahmed, Elsayed Abdelkreem

**Affiliations:** 1 Department of Pediatrics, Faculty of Medicine, Sohag University, Nasser City, Sohag, Egypt; 2 Department of Medical Microbiology and Immunology, Faculty of Medicine, Sohag University, Nasser City, Sohag, Egypt

**Keywords:** *Helicobacter pylori*, Drug-resistant epilepsy, Seizures, Convulsion, Children

## Abstract

**Background:**

*Helicobacter pylori* (*H. pylori*) causes chronic infection in more than half of the population worldwide. Accumulating body of evidence indicates the possible role of *H. Pylori* infection in extra-intestinal health problems, including epilepsy. This study aims to investigate the efficacy of treating *H. pylori* infection on seizure frequency among children with drug-resistant idiopathic generalized epilepsy (IGE).

**Methods:**

A parallel, two-arm, open-label, randomized controlled trial was conducted on 126 children with drug-resistant IGE and positive *H. pylori* stool antigen test who were randomly assigned to study and comparison groups in 1.2:1 ratio. Only the study group received *H. pylori* eradication therapy (esomeprazole, amoxicillin, and clarithromycin) for two weeks. The primary outcome was seizure improvement (≥ 50% seizure frequency reduction compared with baseline) after 2.5 months. Secondary outcomes were occurrence of status epilepticus, escalation of antiseizure medication (ASMs), and adverse effects. Outcomes between the two groups were compared using Chi-square/Fisher exact tests on an intention-to-treat principle. Logistic regression analysis was performed to investigate possible effects of baseline variables on primary outcome.

**Results:**

Seizure improvement occurred in 23 (33%) children in the study group compared with seven (12%) children in the comparison group (Risk ratio [RR] 2.7, 95% confidence interval [CI]: 1.3–5.9; *p* 0.006). The study group had lower occurrence of status epilepticus (2.9% vs. 14%; RR 0.21, 95%CI: 0.05–0.93; *p* 0.042) and lesser need for ASMs escalation (4.4% vs. 19.3%; RR 0.23, 95%CI: 0.07–0.77; *p* 0.010). Adverse effects were more frequent among subjects in the study group, including nausea (15.9% vs. 10.5%) vomiting (8.7% vs. 3.5%), diarrhea (11.6% vs. 5.3%), and skin rash (4.4% vs. 1.8%), but the differences were not statistically significant (*p* > 0.05). None of baseline participants’ variables was significantly associated with the primary outcome.

**Conclusion:**

Treating *H. pylori* infection may improve seizure control in children with drug-resistant IGE, but further studies are warranted to confirm our findings and explore mechanisms behind seizure improvement following *H. pylori* eradication therapy.

**Trial registration:**

Registered on www.clinicaltrials.gov (identifier: NCT05297695) on 17 March 2022. https://clinicaltrials.gov/study/NCT05297695.

**Supplementary Information:**

The online version contains supplementary material available at 10.1186/s13052-025-01956-2.

## Introduction

*Helicobacter pylori* (*H. pylori*) causes chronic infection in more than half of the population worldwide. Most infected children are asymptomatic, but some may develop peptic ulcer disease or rarely cancer [1]. Moreover, an accumulating body of evidence indicates the possible role of *H. Pylori* infection in extra-intestinal health problems, such as iron deficiency anemia, immune thrombocytopenic purpura, Alzheimer disease, Parkinson’s disease, multiple sclerosis, and epilepsy [1–3].

Epilepsy is a common neurological disorder that affects 0.5–1% of all children. The development of epilepsy is highly complex and can be attributed to structural, genetic, infectious, metabolic, and immune factors. However, the etiology remains unknown in about half of epileptic children [4, 5]. Idiopathic generalized epilepsy (IGE) accounts for more than 25% of new-onset epilepsy in children [6]. About one-third of children with epilepsy do not respond well to antiseizure medications (ASMs), which is associated with marked neurobiological, cognitive, psychological, and social consequences [4, 5, 7]. Increased understanding of mechanisms contributing to the development of epilepsy, particularly drug-resistant IGE, could contribute to the advent of new therapeutic strategies to improve patients’ outcomes [8, 9].

Observational studies indicate a higher prevalence of *H. pylori* infection in patients with epilepsy [3, 10–13]. However, the value of treating *H. pylori* infection in patients with epilepsy has been investigated in only a small non-controlled study, which showed no significant change in seizure frequency following *H. pylori* eradication therapy in patients with refractory epilepsy [14].

With this background in mind, we explored the efficacy of treating *H. pylori* infection for pediatric drug-resistant IGE. We hypothesized that among children with drug-resistant IGE, eradication of *H. pylori* infection, compared with no *H. pylori* eradication therapy, is associated with improved seizure control at 2.5 months after drug administration.

## Patients, materials, and methods

### Study design & setting

This parallel, two-arm, superiority, open-label, randomized controlled trial was conducted from April 2022 to December 2023 at the Departments of Pediatrics and Medical Microbiology & Immunology in Sohag University Hospital (Egypt). The study was approved by the Research Ethics Committee of Faculty of Medicine, Sohag University (Approval no. Soh-Med-22-03-13; date 08 March 2022) (additional file 1). Written informed consent was acquired from parents of all participating children. All methods were carried out following the ethical principles contained within the 1964 Declaration of Helsinki and as revised in 2013. The study was prospectively registered on www.clinicaltrials.gov (identifier: NCT05297695) and is reported following the CONSORT guidelines [15]. The CONSORT checklist is provided in the additional file 2.

### Participants

Eligible subjects were children aged between 4 and 18 years who presented with drug-resistant IGE and *H. pylori* infection. IGE was defined following the International League Against Epilepsy (ILAE) guidelines based on typical generalized seizures (absence, myoclonic, or tonic-clonic) and consistent interictal electroencephalography findings (generalized spike-and-wave and/or polyspikes) with no evidence of structural brain abnormalities on magnetic resonance imaging as well as no remarkable interictal neurological manifestations; this includes four well-recognized subtypes–childhood absence epilepsy, juvenile absence epilepsy, juvenile myoclonic epilepsy, and epilepsy with generalized tonic-clonic seizures alone [6, 9]. Drug-resistant epilepsy was defined according to ILAE as failure of proper trials of two tolerated and appropriately selected and used ASMs schedules (whether as mono- or polytherapy) to achieve extended seizure freedom [16]. *H. pylori* infection relied on positive *H. pylori* stool antigen (HpSA) testing using BIOS ENZYME IMMUNOASSAY TEST KIT (Catalog Number: 10224, Chemux BioScience Inc, South San Francisco, CA, USA), following manufacturer’s instructions. Exclusion criteria were failure to obtain informed consent, presence of a clinical indication for treating *H. pylori* infection, known allergy or contraindications to any of the study drugs, and prior treatment with antibiotics and/or proton pump inhibitors in the last two months.

### Sample size calculation

The sample size for this superiority trial was calculated using Stata/BE 17 (StataCorp LLC, College Station, TX, USA), assuming a seizure improvement of 25% in the study group and 5% in the comparison group keeping type 1 error (α) at 0.05 and type II error (β) at 0.2. This resulted in 49 subjects per group, which was raised by 15% for potential dropouts and an additional 20% for possible failure of *H. pylori* eradication therapy in the study group. Accordingly, the final sample size was 69 subjects for the study group and 57 subjects for the comparison group.

### Baseline evaluation

Enrolled children underwent baseline assessment with comprehensive history taking and clinical examination, including sociodemographic data, family history of epilepsy, parental consanguinity, developmental history, perinatal history, details of epilepsy (age at onset, type, monthly seizure frequency as averaged from last two months, status epilepticus in last two months, and ASMs), gastrointestinal problems, anthropometric measures, systematic and detailed neurological examination, and review of investigations.

### Randomization

Participants were randomly assigned to study group or comparison group in 1.2:1 ratio using computer-generated random numbers (randomizer.org). A person outside the study team sealed these random numbers into successively numbered opaque envelopes. For every eligible participant, the envelope in order was opened, and the allocated intervention was implemented. The treating physicians and participants’ parents were aware of the group assignment. Participants’ parents were instructed not to disclose their group assignment to outcome assessors who were blinded to group allocation.

### Intervention

Only the study group received a two-week triple therapy for *H. pylori* infection in the form of esomeprazole (twice daily doses of 20 mg if bodyweight 15–24 kg, 30 mg if bodyweight 25–34 kg, and 40 mg if bodyweight ≥ 35 kg), amoxicillin (twice daily doses of 500 mg if bodyweight 15–24 kg, 750 mg if bodyweight 25–34 kg, and 1000 mg if bodyweight ≥ 35 kg) and clarithromycin (twice daily doses of 250 mg if bodyweight 15–24 kg, 500 mg if bodyweight 25–34 kg, and 500 mg if bodyweight ≥ 35 kg) [17]. Both groups received standard epilepsy care per discretion of their treating physicians. Participants were evaluated after 2.5 months for clinical assessment based on written patient’s diaries and repeating HpSA testing.

### Outcomes

The primary outcome was seizure improvement defined as ≥ 50% seizure frequency reduction (SFR) at 2.5 months following randomization compared with baseline; seizure frequency was estimated as the average monthly seizure frequency in the last two months. The secondary outcomes were the occurrence of status epilepticus, need for escalation of ASMs, and adverse effects of *H. pylori* eradication therapy, including nausea, vomiting, diarrhea, and skin rash.

### Statistical analysis

We analyzed data on an intention-to-treat principle using Stata/BE 17 (StataCorp). Data were expressed as frequencies (%) for categorical data, mean (SD) for normally distributed quantitative data, and median (IQR) for non-normally distributed quantitative data. We compared baseline variables and outcomes between study and comparison groups using Chi-square/Fisher exact tests for categorical data and Student’s t-test/Wilcoxon rank-sum test for quantitative measures, as appropriate. We carried out a sub-group analysis to compare the intervention (*H. pylori* eradication therapy) effect on the primary outcome measure among males/females and those ≤ 10 and > 10 years of age using the Mantel-Haenszel (M-H) method. Furthermore, logistic regression analysis was performed to investigate possible effect of baseline variables on primary outcome measure. We also conducted per-protocol analysis (based on eradication status) of data after exclusion of subjects in the study group who failed *H. pylori* eradication therapy. A *p*-value (two-tailed) < 0.05 was considered statistically significant.

## Results

**Fig. 1:**
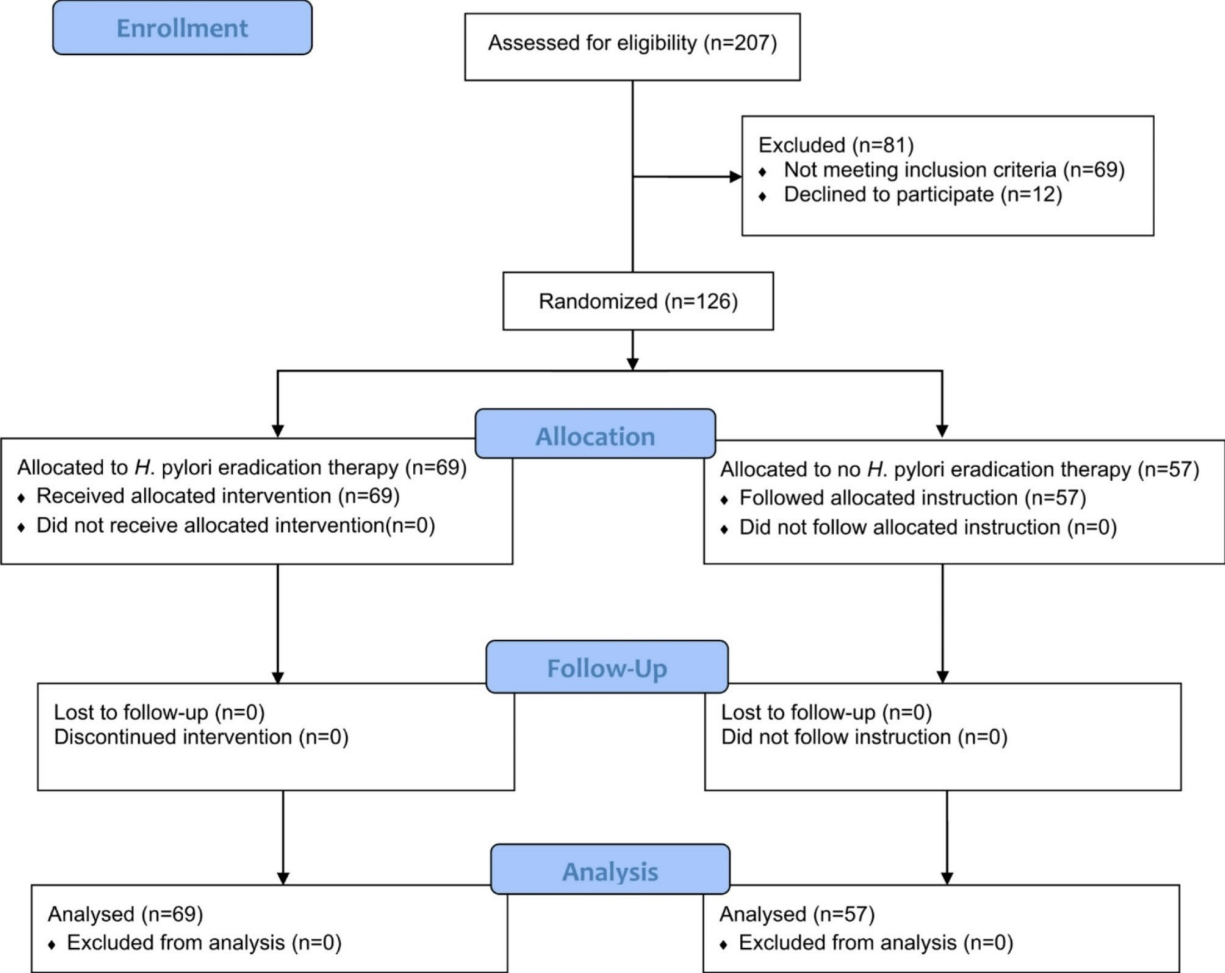
CONSORT flow diagram of the study participants

The CONSORT flow diagram of the study participants is shown in Fig. 1. Out of 207 children assessed for eligibility, 126 were successfully randomized into study (*n* = 69) and comparison (*n* = 57) groups. All participants completed the study, and none were excluded from primary data analysis. The baseline sociodemographic and clinical features of study participants are provided in Table 1. Participants were 82 males and 44 females with a median age of seven years. Most participants (93%) had generalized tonic-clonic seizures. The median frequency of seizures was five per month, and 17.5% of cases experienced at least one episode of status epilepticus in the last two months. Importantly, all baseline variables were comparable between the study and comparison groups.

**Table 1:** Baseline characteristics of study participants

Characteristics	Total (*n* = 126)	Study group (*n* = 69)	Comparison group (*n* = 57)	*p*-value
Age (years)^*^	7 (5–10)	7 (5–11)	7 (5.5-9)	0.961
Male sex^#^	82 (65.1%)	44 (63.8%)	38 (66.7%)	0.734
Body mass index (kg/m^2^)^$^	16.5 (2.89)	16.5 (3.25)	16.6 (2.41)	0.777
Head circumference (cm)^$^	50.4 (1.30)	50.3 (1.42)	50.6 (1.13)	0.225
Urban residence^#^	32 (25.4%)	16 (23.2%)	16 (28.1%)	0.531
Low socioeconomic level^#^	71 (56.4)	39 (56.5%)	32 (56.1%)	0.966
Low parental education^#^	88 (69.8%)	49 (71.0%)	39 (68.4%)	0.752
Parental work^#^				0.195
None	17 (13.5%)	10 (14.5%)	7 (12.3%)	
Government	62 (49.2%)	29 (42.0%)	33 (57.9%)	
Private	47 (37.3%)	30 (43.5%)	17 (29.8%)	
Parental consanguinity^#^	85 (67.5%)	43 (62.3%)	42 (73.7%)	0.175
Family history of epilepsy^#^	26 (20.6%)	16 (23.2%)	10 (17.5%)	0.436
Gastrointestinal manifestations^#^	61 (48.4%)	32 (46.4%)	29 (50.9%)	0.615
Seizures, generalized tonic-clonic/absence	117/9	63/6	54/3	0.511
Seizure frequency per month^*^	5 (3–6)	5 (3–6)	4 (3–6)	0.813
Status epilepticus in last 2 months^#^	22 (17.5%)	12 (17.4%)	10 (17.5%)	0.982
Anti-seizure medication^#^				
Levetiracetam	126 (100%)	69 (100%)	57 (100%)	NA
Sodium valproate	124 (98.4%)	68 (98.6%)	56 (98.3%)	1.000
Topiramate	66 (52.4%)	34 (49.3%)	32 (56.1%)	0.443
Clonazepam	31 (24.6%)	18 (26.1%)	13 (22.8%)	0.670

^*^ median (IQR), ^#^ number (%), ^$^ mean (SD)

Data were analyzed using Student t-/Mann-Whitney tests for continuous data and Pearson Chi-Square/Fisher’s Exact tests for categorical data

The study outcomes of primary (intention-to-treat) analysis are shown in Table 2. At 2.5 months after randomization, seizure improvement occurred in 23 (33%) children in the study group compared with seven (12%) children in the comparison group (Risk ratio [RR] 2.7, 95% confidence interval [CI]: 1.3–5.9; *p* 0.006]; number needed to treat is 4.8. Additionally, the study group had lower occurrence of status epilepticus (2.9% vs. 14%; RR 0.21, 95%CI: 0.05–0.93; *p* 0.042) and lesser need for ASMs escalation (4.4% vs. 19.3%; RR 0.23, 95%CI: 0.07–0.77; *p* 0.010]. Adverse effects were more frequent among subjects in the study group, including nausea (15.9% vs. 10.5%) vomiting (8.7% vs. 3.5%), diarrhea (11.6% vs. 5.3%), and skin rash (4.4% vs. 1.8%), but the differences did not reach statistically significant levels.

**Table 2:** Outcomes between study groups

Outcomes	Study group (*n* = 69)	Comparison group (*n* = 57)	Risk Ratio (95%CI)	*p*-value
Improved seizures (≥ 50% SFR)	23 (33.3%)	7 (12.3%)	2.7 (1.3–5.9)	0.006
Status epilepticus	2 (2.9%)	8 (14.0%)	0.21 (0.05–0.93)	0.042
Escalation of ASMs	3 (4.4%)	11 (19.3%)	0.23 (0.07–0.77)	0.010
Nausea	11 (15.9%)	6 (10.5%)	1.5 (0.60–3.8)	0.376
Vomiting	6 (8.7%)	2 (3.5%)	2.5 (0.52–11.8)	0.292
Diarrhea	8 (11.6%)	3 (5.3%)	2.2 (0.61–7.9)	0.342
Skin rash	3 (4.4%)	1 (1.8%)	2.5 (0.27–23.2)	0.626

Data are presented as number (%) and analyzed using Pearson Chi-Square/Fisher’s Exact tests

ASMs, anti-seizure medications; CI, confidence interval; SFR, seizure frequency reduction

Subgroup analysis revealed that *H. pylori* eradication therapy results in more frequent seizure improvement (primary outcome) among males and those older than 10 years, but these differences did not reach statistically significant levels (additional file 3). Furthermore, univariate logistic regression revealed that none of baseline patients’ variables was significantly associated with the primary outcome (additional file 4). In contrast, *H. Pylori* eradication therapy was associated with 3.6 higher odds of having seizure improvement.

We performed a per-protocol analysis based on *H. pylori* eradication status by excluding 10 subjects from the study group who remained positive for HpSA testing, indicating failure of *H. pylori* eradication therapy. The results were generally similar to that of primary analysis with more pronounced efficacy outcomes between study and comparison groups with regard to seizure improvement (37% vs. 12%; RR 3.0, 95%CI: 1.4–6.6; *p* 0.002), status epilepticus (1.7% vs. 14%; RR 0.12, 95%CI: 0.02–0.94; *p* 0.016), and ASMs escalation (3.4% vs. 19.3%; RR 0.18, 95%CI: 0.04–0.76; *p* 0.008) (Table 3). Likewise, the patients’ baseline variables were comparable between the study and comparison groups (additional file 5), and none of these baseline variables has significant association with the primary outcome (additional file 6).

**Table 3:** Per-protocol analysis of outcomes between study groups*

Outcomes	Study group (*n* = 59)	Comparison group (*n* = 57)	Risk Ratio (95%CI)	*p*-value
Improved seizures (≥ 50% SFR)	22 (37.3%)	7 (12.3%)	3.0 (1.4–6.6)	0.002
Status epilepticus	1 (1.7%)	8 (14.0%)	0.12 (0.02–0.94)	0.016
Escalation of ASMs	2 (3.4%)	11 (19.3%)	0.18 (0.04–0.76)	0.008
Nausea	7 (11.9%)	6 (10.5%)	1.1 (0.40–3.2)	0.819
Vomiting	5 (8.5%)	2 (3.5%)	2.4 (0.49-12)	0.439
Diarrhea	6 (10.2%)	3 (5.3%)	1.9 (0.51–7.4)	0.491
Skin rash	3 (5.1%)	1 (1.8%)	2.9 (0.31-27)	0.619

*After exclusion of 10 cases from study group who failed *Helicobacter pylori* eradication therapy

Data are presented as number (%) and analyzed using Pearson Chi-Square/Fisher’s Exact tests

ASMs, anti-seizure medications; CI, confidence interval; SFR, seizure frequency reduction

## Discussion

This is the first randomized-controlled study to investigate the efficacy of treating *H. pylori* infection on seizure frequency in children with drug-resistant IGE. We found that *H. pylori* eradication therapy leads to significant improvement in seizures (≥ 50% SFR); on average, five patients would have to receive *H. pylori* eradication therapy for one additional patient to have improved seizure control. Moreover, eradicating *H. pylori* infection resulted in a lower occurrence of status epilepticus and lower need for escalation of ASMs with no significant adverse effects. This indicates that eradicating *H. pylori* infection could be a promising therapeutic option to control seizures in children with drug-resistant IGE, but further confirmatory studies are warranted.

Our study findings are in disagreement with Asadi-Pooya et al. [14] who reported that treating *H. pylori* infection is not significantly associated with change of seizure frequency in patients with refractory epilepsy. However, Asadi-Pooya et al. [14] study is markedly underpowered with too small sample size of only nine patients, and it lacks a control group, which raises concerns about possible selection bias and confounding. Furthermore, our study included only patients with IGE, while Asadi-Pooya et al. [14] mostly included patients with temporal lobe epilepsy; it is possible that *H. pylori* eradication therapy is more effective in patients with IGE than other epilepsy types, but this postulation remains to be confirmed by future studies.

Observational studies on the association between *H. pylori* infection and epilepsy have shown inconsistent results. Okuda et al. [10] and Ozturk et al. [11] reported a significantly higher prevalence of *H. pylori* infection among patients with epilepsy, and that *H. pylori* infection is associated with worse prognosis. In contrast, Asadi-Pooya et al. [12] showed no significant difference in prevalence of *H. pylori* infection between adult epileptic patients and healthy controls. However, the healthy control group was older than both epileptic groups, which could confound the results since *H. pylori* prevalence is higher among older individuals [18]. Moreover, given the reported high prevalence of *H. pylori* infection, a higher number of participants would be required for an adequately powered study. In another study, adult epileptic patients had more prevalent *H. pylori* infection than healthy controls (64.3% vs. 35.7%), but the difference did not reach statistical significance likely due to the small sample size [13]. A meta-analysis of these four observational studies concluded that the prevalence of *H. pylori* infection is significantly higher among epileptic patients than healthy population (46.98% vs. 26.34%, OR 2.58) [3].

The association between *H. pylori* infection and epilepsy may be attributed to several biological mechanisms. First, a cross-mimicry between *H. pylori* and human cellular phospholipids may result in *H. pylori*-induced autoimmune reaction with production of anticardiolipin antibodies [10]. Studies have shown that patients with epilepsy, particularly idiopathic epilepsy, have an increased prevalence of autoantibodies, including anticardiolipin antibodies [19–21]. Importantly, one study reported that *H. pylori* eradication therapy is associated with gradual resolution of neurological symptoms in a patient with antiphospholipid antibody syndrome [22]. Another possible mechanism is *H. pylori* infection-induced low-grade systemic inflammation with local secretion of proinflammatory mediators (e.g., interleukin-8,-6, -1beta, -10-12, tumor necrosis factor, interferon gamma) [2, 23]. Persistent release of proinflammatory mediators into the systemic circulation may induce disruption of blood-brain barrier, neuroinflammation, and neurotoxicity, all of which contribute to the pathogenesis of epilepsy and lowering of seizure threshold [3, 18, 23]. Additionally, *H. pylori* infection may induce the release of multiple neurotransmitters, such as serotonin, dopamine, acetylcholine, adrenaline, and noradrenaline [24]. Furthermore, *H. pylori* infection may lead to neuronal/axonal damage, formation of free radicals, and changes in the expression of neuropeptides, such as vasoactive intestinal peptide and c-fos [24]. Last, *H. pylori* infection may induce alterations in the composition of gut microbiome, which may be associated with poor response of epileptic patients to ASMs [25–28].

Of note, it is possible that *H. pylori* eradication therapy might help control seizures in children with drug-resistant IGA by mechanisms other than eradication of *H. pylori* infection. For instance, clarithromycin has the potential to inhibit the metabolism and increase serum levels of carbamazepine [29]. Additionally, proton pump inhibitors exert antioxidant, anti-inflammatory, and anti-apoptotic effects, which may play a protective role in the pathogenesis of epilepsy and neurodegenerative diseases [30]. Of note, pantoprazole has been shown to significantly improve seizures in pentylenetetrazole‑induced epileptic seizures in rats [31].

Our findings go in line with studies showing that treating *H. pylori* infection improves clinical outcomes of patients with certain neurological disorders, such as Parkinson disease and Alzheimer’s disease, which emphasizes the microbial-neurological connection and the gut-brain interface [23, 32, 33]. Given that *H. pylori* infects more than half of population worldwide, further studies are required to investigate its role in the pathogenesis of neurological and seizure disorders and the potential value of *H. pylori* eradication therapy [23, 29].

The key strengths of the current study include its randomized controlled study design, conducting both intention-to-treat and per-protocol analyses, and the focus on meaningful clinical outcomes. However, we acknowledge some study limitations. First, the relatively small sample size might preclude study power to detect some statistically significant associations. Moreover, while the outcome assessors were blinded to study group allocation, the lack of blinding for participants and treating physicians leaves the room for potential bias. In addition, *H. pylori* infection was not confirmed by endoscopic biopsy and histopathological examination. The diagnosis of *H. pylori* infection was based on HpSA testing, which is a widely available and cost-effective technique for primary diagnosis and eradication monitoring with sensitivity and specificity of 95% and 97.6%, respectively [1, 2]. While we can not absolutely exclude the possibility that some participants might have false positive HpSA testing, this is unlikely to affect study findings since such possible participants with false positive results would be randomly distributed between the study and comparison group, and such non-differential misclassification might result in biasing the risk ratio towards rather than away from the null. However, more specific diagnostic tests for *H. pylori* infection are certainly recommended in future studies. Another limitation is related to the lack of extensive laboratory or neurophysiological studies to unravel the exact pathophysiological mechanisms for seizure improvement following *H. pylori* eradication therapy. Future studies should delve deeper into these mechanisms (e.g., assessing inflammatory markers, gut microbiome analysis, neuroimaging studies) to explain how *H. pylori* interacts with neurological health and predisposes to epilepsy. Furthermore, the short follow-up period (2.5 months) is not enough to determine whether the observed benefits of *H. pylori* eradication are sustainable over the long-term, which is particularly important when considering epilepsy as a chronic condition that demands consistent management. Finally, this study included children with drug-resistant IGE from a single center in Egypt. Accordingly, the findings may not be generalizable to populations from different regions or with varied dietary and microbiome profiles, which could be addressed by a broader and multi-center approach in future studies.

## Conclusion

Treating *H. pylori* infection may lead to improvement of seizures in children with drug-resistant idiopathic generalized epilepsy. Future studies should include double-blind design, extended follow-up period, and exploring biological mechanisms behind improved seizures following *H. pylori* eradication therapy.

## Electronic supplementary material

Below is the link to the electronic supplementary material.

**Additional file 1** Study approval from the Research Ethics Committee

**Additional file 2** CONSORT 2010 checklist

**Additional file 3** Stratified analysis by age and sex

**Additional file 4** Univariate analysis for predictors of improved seizures (≥ 50% seizure frequency reduction) in children with drug-resistant idiopathic generalized epilepsy (*n* = 126)

**Additional file 5** Per-protocol analysis of baseline characteristics of study participants

**Additional file 6** Per-protocol univariate analysis for predictors of seizure improvement (≥ 50% seizure frequency reduction) in children with drug-resistant idiopathic generalized epilepsy (*n* = 116)

## Data Availability

The data that support the study findings are available from the corresponding author on reasonable request.

## Abbreviations

ASMs: Antiseizure medication
CI: Confidence interval
H. pylori: Helicobacter pylori
HpSA: H. pylori stool antigen
IGE: Idiopathic generalized epilepsy
ILAE: International League Against Epilepsy
RR: Risk ratio
SFR: Seizure frequency reduction
