# Supplementary material for: Efficacy of treating Helicobacter pylori infection on seizure frequency in children with drug-resistant idiopathic generalized epilepsy: a randomized controlled trial
Source: Ital J Pediatr. 2025 Apr 17;51:121. doi: 10.1186/s13052-025-01956-2 (PMC12004564; doi:10.1186/s13052-025-01956-2)
Supplement: Supplementary file 3 — Additional file 3 Stratified analysis by age and sex [file 13052_2025_1956_MOESM3_ESM.docx]

**Additional file 3** Stratified analysis by age and sex

**Table 1.** Improved seizures (≥ 50% seizure frequency reduction) at 2.5 months after randomization by age

| **Age groups** | **Study group**  (*n*=69) | **Comparison group**  (*n*=57) |
| --- | --- | --- |
| ≤10 years (*n*=101) | 15/36 (29.4%) | 6/44 (12.0%) |
| >10 years (*n*=25) | 8/10 (44.4%) | 1/6 (16.7%) |

Data are presented as number (%)

CI, confidence interval

Mantel-Haenszel risk ratio (95%CI): crude=2.7 (1.3, 5.9); combined=2.7 (1.2, 6.2); *p* 0.307

**Table 2.** Improved seizures (≥ 50% seizure frequency reduction) at 2.5 months after randomization by sex

| **Sex** | **Study group**  (*n*=69) | **Comparison group**  (*n*=57) |
| --- | --- | --- |
| Male (*n*=82) | 16/28 (36.4%) | 4/34 (10.5%) |
| Females (*n*=44) | 7/18 (28.0%) | 3/16 (15.8%) |

Data are presented as number (%)

CI, confidence interval;

Mantel-Haenszel risk ratio (95%CI): crude=2.7 (1.3, 5.9); combined=2.7 (1.3, 5.8); *p* 0.405
