# Supplementary material for: Efficacy of treating Helicobacter pylori infection on seizure frequency in children with drug-resistant idiopathic generalized epilepsy: a randomized controlled trial
Source: Ital J Pediatr. 2025 Apr 17;51:121. doi: 10.1186/s13052-025-01956-2 (PMC12004564; doi:10.1186/s13052-025-01956-2)
Supplement: Supplementary file 4 — Additional file 4 Univariate analysis for predictors of improved seizures (≥ 50% seizure frequency reduction) in children with drug-resistant idiopathic generalized epilepsy (n = 126) [file 13052_2025_1956_MOESM4_ESM.docx]

**Additional file 4** Univariate analysis for predictors of improved seizures (≥ 50% seizure frequency reduction) in children with drug-resistant idiopathic generalized epilepsy (*n*=126)

| **Characteristics** | **Odds ratio** (95%CI) | ***p*-value** |
| --- | --- | --- |
| Age (years) | 1.01 (0.92-1.15) | 0.916 |
| Male (vs. female) | 1.10 (0.46-2.61) | 0.835 |
| Body mass index (kg/m^2^) | 1.01 (0.87-1.16) | 0.951 |
| Head circumference (cm) | 0.81 (0.588, 1.13) | 0.212 |
| Urban residence (vs. rural) | 0.86 (0.33, 2.26) | 0.766 |
| Low socioeconomic level (vs. middle/high) | 1.02 (0.45-2.33) | 0.968 |
| Low parental education (vs. middle/high) | 0.83 (0.34-1.98) | 0.665 |
| Parental work |  |  |
| None | Ref |  |
| Government | 1.04 (0.30-3.67) | 0.955 |
| Private | 0.99 (0.27-3.67) | 0.992 |
| Parental consanguinity | 1.44 (0.58, 3.59) | 0.433 |
| Family history of epilepsy | 0.95 (0.34-2.64) | 0.922 |
| Gastrointestinal manifestations | 1.55 (0.68-3.53) | 0.302 |
| Generalized tonic-clonic seizures (vs. absence) | 0.36 (0.09-1.43) | 0.145 |
| Seizure frequency per month | 1.15 (0.95-1.39) | 0.154 |
| Status epilepticus in last 2 months | 0.45 (0.12-1.64) | 0.227 |
| Anti-seizure medications |  |  |
| Levetiracetam (vs. others) | NA |  |
| Sodium valproate (vs. others) | 0.31 (0.02-5.03) | 0.407 |
| Topiramate (vs. others) | 1.5 (0.65-3.45) | 0.340 |
| Clonazepam (vs. others) | 1.44 (0.58-3.60) | 0.433 |
| *H. pylori* eradication therapy | 3.57 (1.40-9.11) | 0.008 |

CI, confidence interval; *H. Pylori; Helicobacter pylori*
