# Supplementary material for: Efficacy of treating Helicobacter pylori infection on seizure frequency in children with drug-resistant idiopathic generalized epilepsy: a randomized controlled trial
Source: Ital J Pediatr. 2025 Apr 17;51:121. doi: 10.1186/s13052-025-01956-2 (PMC12004564; doi:10.1186/s13052-025-01956-2)
Supplement: Supplementary file 5 — Additional file 5 Per-protocol analysis of baseline characteristics of study participants [file 13052_2025_1956_MOESM5_ESM.docx]

**Additional file 5** Per-protocol analysis of baseline characteristics of study participants**^¶^**

| **Characteristics** | **Total**  (*n*=116) | **Study group**  (*n*=59) | **Comparison group**  (*n*=57) | ***p-*value** |
| --- | --- | --- | --- | --- |
| Age (years)**^*^** | 7 (5-10) | 7 (5-11) | 7 (5.5-9) | 0.987 |
| Male sex**^#^** | 75 (64.7%) | 37 (62.7%) | 38 (66.7%) | 0.656 |
| Body mass index (kg/m^2^)**^$^** | 16.6 (2.91) | 16.6 (3.35) | 16.6 (2.41) | 0.921 |
| Head circumference (cm)**^$^** | 50.5 (1.29) | 50.3 (1.43) | 50.6 (1.13) | 0.270 |
| Urban residence**^#^** | 31 (26.7%) | 15 (25.4%) | 16 (28.1%) | 0.747 |
| Low socioeconomic level**^#^** | 62 (53.5%) | 30 (50.9%) | 32 (56.1%) | 0.568 |
| Low parental education**^#^** | 80 (69.0%) | 41 (69.5%) | 39 (68.4%) | 0.901 |
| Parental work**^#^** |  |  |  | 0.241 |
| None | 16 (13.8%) | 9 (15.3%) | 7 (12.3%) |  |
| Government | 58 (50.0%) | 25 (42.4%) | 33 (57.9%) |  |
| Private | 42 (36.2%) | 25 (42.4%) | 17 (29.8%) |  |
| Parental consanguinity**^#^** | 79 (68.1%) | 37 (62.7%) | 42 (73.7%) | 0.205 |
| Family history of epilepsy**^#^** | 25 (21.6%) | 15 (25.4%) | 10 (17.5%) | 0.302 |
| Gastrointestinal manifestations**^#^** | 55 (47.4%) | 26 (44.1%) | 29 (50.9%) | 0.463 |
| Seizures, generalized tonic-clonic/absence | 109/7 | 55/4 | 54/3 | 1.000 |
| Seizure frequency per month**^*^** | 5 (4-6) | 5 (4-6) | 4 (3-6) | 0.326 |
| Status epilepticus in last 2 months**^#^** | 22 (19.0%) | 12 (20.3%) | 10 (17.5%) | 0.701 |
| Anti-seizure medications**^#^** |  |  |  |  |
| Levetiracetam | 116 (100%) | 59 (100%) | 57 (100%) | NA |
| Sodium valproate | 114 (98.3%) | 58 (98.3%) | 56 (98.3%) | 1.000 |
| Topiramate | 61 (52.6%) | 29 (49.2%) | 32 (56.1%) | 0.451 |
| Clonazepam | 29 (25.0%) | 16 (27.1%) | 13 (22.8%) | 0.592 |

**^¶^**After exclusion of 10 cases from study group who failed *Helicobacter* pylori eradication therapy

**^*^** median (IQR), **^#^** number (%), **^$^** mean (SD)

Data were analyzed using Student t-/Mann-Whitney tests for continuous data and Pearson Chi-Square/Fisher’s Exact tests for categorical data
