# Supplementary material for: Efficacy of treating Helicobacter pylori infection on seizure frequency in children with drug-resistant idiopathic generalized epilepsy: a randomized controlled trial
Source: Ital J Pediatr. 2025 Apr 17;51:121. doi: 10.1186/s13052-025-01956-2 (PMC12004564; doi:10.1186/s13052-025-01956-2)
Supplement: Supplementary file 6 — Additional file 6 Per-protocol univariate analysis for predictors of seizure improvement (≥ 50% seizure frequency reduction) in children with drug-resistant idiopathic generalized epilepsy (n = 116) [file 13052_2025_1956_MOESM6_ESM.docx]

**Additional file 6** Per-protocol univariate analysis for predictors of seizure improvement (≥ 50% seizure frequency reduction) in children with drug-resistant idiopathic generalized epilepsy (*n*=116)^*^

| **Characteristics** | **Odds ratio** (95%CI) | ***p*-value** |
| --- | --- | --- |
| Age (years) | 0.99 (0.87-1.14) | 0.923 |
| Male (vs. female) | 1.05 (0.44-2.54) | 0.911 |
| Body mass index (Kg/m^2^) | 1.01 (0.88-1.17) | 0.855 |
| Head circumference (cm) | 0.75 (0.53-1.06) | 0.102 |
| Urban residence (vs. rural) | 0.84 (0.32-2.21) | 0.717 |
| Low socioeconomic level (vs. middle/high) | 1.10 (0.47-2.55) | 0.830 |
| Low parental education (vs. middle/high) | 0.81 (0.33-1.98) | 0.643 |
| Parental work |  |  |
| None | Ref |  |
| Government | 1.05 (0.29-3.75) | 0.944 |
| Private | 0.94 (0.25-3.75) | 0.925 |
| Parental consanguinity | 1.31 (0.52-3.32) | 0.566 |
| Family history of epilepsy | 0.93 (00.33-2.62) | 0.896 |
| Gastrointestinal manifestations | 1.52 (0.65-3.53) | 0.335 |
| Generalized tonic-clonic (vs. absence) | 0.42 (0.09-2.0) | 0.273 |
| Seizure frequency per month | 1.10 (0.91-1.34) | 0.330 |
| Status epilepticus in last 2 months | 0.41 (0.11-1.51) | 0.182 |
| Anti-seizure medications |  |  |
| Levetiracetam (vs. others) | NA |  |
| Sodium valproate (vs. others) | 0.33 (0.02-5.38) | 0.433 |
| Topiramate (vs. others) | 1.39 (0.59-3.24) | 0.453 |
| Clonazepam (vs. others) | 1.19 (0.46-3.10) | 0.711 |
| *H. pylori* eradication therapy | 4.25 (1.64-11) | 0.003 |

*After exclusion of 10 cases from study group who failed Helicobacter pylori eradication therapy

CI, confidence interval; *H. pylori; Helicobacter pylori*
