# Supplementary material for: Efficacy of treating Helicobacter pylori infection on seizure frequency in children with drug-resistant idiopathic generalized epilepsy: a randomized controlled trial
Source: Ital J Pediatr. 2025 Apr 17;51:121. doi: 10.1186/s13052-025-01956-2 (PMC12004564; doi:10.1186/s13052-025-01956-2)
Supplement: Supplementary file 1 — Additional file 1 Study approval from the Research Ethics Committee [file 13052_2025_1956_MOESM1_ESM.pdf]

Medical Research Ethics Committee
Faculty of Medicine - Sohag University
OHRP #: IRB00013006

Dear Dr. / ***Mina Samy Basily,*** Department of *Pediatrics*

As regards, the request for reviewing your proposed *Master Degree* research protocol under the title of: "*Effect of Treating Helicobacter Pylori Infection on Seizure Frequency in Children with Drug-Resistant Idiopathic Generalized Epilepsy*", we are pleased to inform you that the Medical Research Ethics Committee (MREC) of the Faculty of Medicine, Sohag University has reviewed the proposed protocol and approved it according to the committee standard operating procedure guidelines on 08 /3 /2022 under ***IRB*** Registration number: ***Soh-Med-22-03-13***

Please be advised that any changes in this accepted proposal should be notified.

With best regards,

Head of committee

Tarek Abulezz

Medical Research Ethics Committee
Sohag University
Faculty of Medicine

Printed on 09 /03 /2022
